# Stronger Antipredatory Vigilance of Prey to Olfactory Cues From Injured Vulnerable Conspecifics

**DOI:** 10.1002/ece3.72257

**Published:** 2025-10-06

**Authors:** Resona Simkhada, Jhaman Kundun, Svetla Sofkova‐Bobcheva, Xiong Zhao He

**Affiliations:** ^1^ School of Agriculture and Environment Massey University Palmerston North New Zealand; ^2^ Nepal Agricultural Research Council Singhadurbarplaza, Kathmandu Nepal; ^3^ Caribbean Agricultural Research and Development Institute Mon Repos East Coast Demerara Guyana

**Keywords:** antipredatory vigilance, life stage‐specific vulnerability, non‐consumption effect, offspring sex ratio, olfactory cue, reproduction

## Abstract

Predation risk is a key evolutionary force shaping prey behaviors and life‐history strategies across taxa. Predators often target vulnerable life stages of prey, but how prey females adjust their reproductive strategies in response to cues from injured conspecifics of these stages remains unclear, particularly in haplodiploid species, where mothers can adjust offspring sex ratios in response to social environments. Using the predatory mite *Phytoseiulus persimilis* and its prey, the spider mite *Tetranychus ludeni*, we first investigated the stage‐specific vulnerability by exposing *T. ludeni* eggs, deutonymphs, and female adults to *P. persimilis* for choice. We then tested whether ovipositing *T. ludeni* females adjusted reproductive performances and survival when exposed to potential predatory cues from those injured conspecifics. Results show that *P. persimilis* significantly preferred *T. ludeni* eggs for feeding, indicating their higher vulnerability to predators. *T. ludeni* females responded most strongly to potential predatory cues from injured eggs, reducing fecundity and producing smaller eggs, but without trading off their longevity. Additionally, when exposed to injured adult cues, *T. ludeni* females adjusted offspring sex ratios, producing more dispersing daughters by fertilizing more smaller eggs, an evolved strategy to escape from the risky environments. In contrast, egg hatching and immature survival were unaffected by conspecific cues. Our results demonstrate that *T. ludeni* females may discriminate among cues from injured conspecifics of different life stages, with the strongest vigilance elicited by cues from the most vulnerable stage (i.e., eggs). This study highlights the role of indirect, life stage‐specific cues in shaping antipredator strategies and reveals that non‐consumptive effects of predation risk could influence prey population dynamics in ways beyond direct predation. Our findings provide a mechanistic understanding of reproductive plasticity in haplodiploid systems, offering new insights into how prey balance current and future reproductive investments under predation pressure.

## Graphical abstract

Eggs of spider mites are more vulnerable to predators. Reproductive females of spider mites are more vigilant to olfactory cues from injured eggs compared to that from injured adults and deutonymphs.

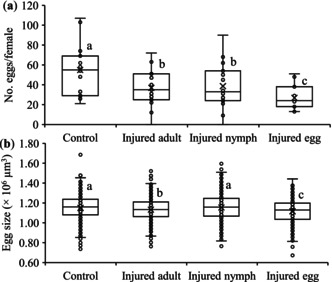

## 1 Introduction

Predation is a fundamental force driving animal evolution (Darwin 1871), and how animals manage predation risk is one of the most studied topics in behavioral ecology. For many species, vulnerability to predation varies between life stages, with early or juvenile life stages typically at higher risk due to their smaller size, lack of antipredator experience, and undeveloped physical or behavioral defenses (Lingle et al. 2008; Choh et al. 2012; Giachetti et al. 2022; Pringle et al. 2019). Consequently, predators prefer these vulnerable life stages due to their higher profitability and lower risk of injury (Giachetti et al. 2022), driving evolutionary and ecological feedback (Mitchell and Harborne 2020). So far, much research focuses on juvenile susceptibility and adult antipredator strategies (e.g., Lima 2009; Choh et al. 2012; Clinchy et al. 2013; de Almeida and Janssen 2013; Panday et al. 2021; Giachetti et al. 2022), and few studies have examined how reproducing females respond to indirect predation cues, particularly when exposed to artificially injured conspecifics that simulate natural predation pressure (Grostal and Dicke 1999; Ferrari et al. 2010; Corbel and Carazo 2022; Ristyadi et al. 2022). Given that maternal stress can influence current reproduction and offspring fitness (Škaloudová et al. 2007; Sheriff et al. 2009; McGhee et al. 2012; Creel et al. 2013; Bell et al. 2016), understanding these responses is crucial for elucidating the broader ecological and evolutionary consequences of predation risk across life stages with varying vulnerability.

Animals employ diverse antipredator strategies to minimize or even avoid predatory threats through behavioral, physiological, and/or morphological responses (Hackl and Schausberger 2014; Hettyey et al. 2015; Dias et al. 2016; Dittmann and Schausberger 2017; Palmer and Packer 2021; Haberkern et al. 2024). They assess risks by perceiving the direct predator‐borne cues (e.g., predator odors and kairomones) and indirect prey‐borne cues (e.g., conspecific alarm signals and damage‐released chemicals) (Thorson et al. 1998; Grostal and Dicke 1999; Fievet et al. 2008; Van Buskirk et al. 2011; Hettyey et al. 2015; Gyuris et al. 2017; Jones et al. 2024). Previous research provided evidence that indirect cues may provide more general information about risk (Thorson et al. 1998; Ehlman et al. 2019; Orrock et al. 2004; Grason and Miner 2012; Grason 2017) and are more reliable than direct cues (Schmidt et al. 2008; Pereira et al. 2017; Arvigo et al. 2019). Many species rely heavily on indirect cues of conspecific injury to assess predation risk (Peacor 2003; Orrock et al. 2004), even in the absence of direct predatory cues (Grostal and Dicke 1999; Bryer et al. 2001; Gyuris et al. 2017; Ristyadi et al. 2022). However, whether prey can discern the life‐stage origin of these cues and adjust reproductive strategies accordingly remains unexplored. Such abilities are essential for prey because stage‐specific vulnerability likely shapes the fitness consequences of maternal decisions, particularly in species with complex life histories.

Spider mites in the genus *Tetranychus* (Acari: Tetranychidae) are group‐living species (Strong et al. 1997; Dhooria 2016; Schausberger et al. 2021), cooperating in host plant colonization and exploitation, oviposition, and dispersal (Schausberger et al. 2021). However, like other small insects with soft bodies such as thrips, whiteflies, psyllids, and aphids (e.g., Cuthbertson et al. 2003; Abou Jawdah et al. 2024; Cardoso et al. 2025; Le Hesran et al. 2025), spider mites are subject to predation by many predators, for example, the predatory mites (Acari: Phytoseiidae) (e.g., Grostal and Dicke 1999; Li and Zhang 2019; Ristyadi et al. 2022). They are vigilant to predation risks, and their decisions regarding habitat selection, reproduction, and dispersal in spider mites are closely tied to social and environmental cues such as the direct predator‐borne cues (Oku and Yano 2007; Lemos et al. 2010; Bowler et al. 2013; Otsuki and Yano 2014a, 2014b; Dittmann and Schausberger 2017; Schausberger et al. 2021). For example, upon detecting the presence of predators, spider mite females will increase locomotion activity (Grostal and Dicke 1999), reduce investment in reproduction (Choh et al. 2010; Li and Zhang 2019; Ristyadi et al. 2022), prolong immature development (Li and Zhang 2019), shorten female longevity (Li and Zhang 2019; Ristyadi et al. 2022), leave patches containing predators (Grostal and Dicke 1999; Pallini et al. 1999; Oku et al. 2004; Bowler et al. 2013), aggregate more tightly (Dittmann and Schausberger 2017), or shift their oviposition sites onto the silk webs to decrease the probability of egg predation (Oku and Yano 2007; Lemos et al. 2010; Otsuki and Yano 2017).

As reported in other animals (Smith 1989; Wudkevich et al. 1997), spider mites exposed to cues from injured conspecifics also display antipredator behaviors (Gyuris et al. 2017) including avoiding leaves with injured conspecifics (Grostal and Dicke 1999) and taking refuge on or in the webbing for reproduction (Oku, Yano, Osakabe, and Takafuji 2003; Oku, Yano, and Takafuji 2003). Gyuris et al. (2017) further demonstrated that in *T. urticae* , reproducing females respond to the olfactory cues originating from injured conspecifics as strongly as their responses to visual and chemical cues, indicating the critical role of olfactory cues in the antipredator response of spider mites. It is well known that although phytoseiid mites can feed on all life stages of tetranychid mites, many species strongly prefer eggs and larvae over nymphs and adults (e.g., Blackwood et al. 2001; Badii et al. 2004; Furuichi et al. 2005; Ganjisaffar and Perring 2015; Jyothis and Ramani 2024). Nonetheless, previous research usually focused on adult‐derived cues (Grostal and Dicke 1999; Oku, Yano, Osakabe, and Takafuji 2003; Oku, Yano, and Takafuji 2003; Ristyadi et al. 2022). Whether ovipositing females can accurately perceive and assess chemical cues from injured conspecifics of different life stages and how they adjust their reproductive strategies is largely unknown.

*Tetranychus ludeni*, native to Europe, has now invaded all continents except Antarctica (Migeon et al. 2010; Zhou et al. 2021a; Migeon and Dorkeld 2024). It attacks more than 370 plant species (Migeon and Dorkeld 2024) including many economically important crops in the warm regions and greenhouses of temperate areas (Zhang 2002; Migeon and Dorkeld 2024). The predatory mite *Phytoseiulus persimilis* Athias‐Henriot (Acari: Phytoseiidae) is among the most extensively studied and widely utilized in biological control programs, especially for spider mite management (Hagen et al. 1999; Tiftikçi et al. 2020; Zhao et al. 2023). In this study, we employed the *P. persimilis*–*T. ludeni* system to assess the vulnerability of different *T. ludeni* life stages to *P. persimilis* predation and investigate the vigilance of ovipositing *T. ludeni* females to olfactory cues from injured conspecifics of different life stages. We first exposed *T. ludeni* of different life stages to *P. persimilis* for choice to determine the vulnerability of *T. ludeni*. We then exposed the *T. ludeni* females to the olfactory cues from the injured conspecific eggs, deutonymphs, or female adults, and subsequently monitored the reproductive outputs and survival. Our study bridges predator–prey behavioral ecology and maternal responses to predatory stresses, delivering insights into the role of indirect cues in shaping antipredator strategies.

## 2 Materials and Methods

### 2.1 Mite Colonies and Environmental Conditions

We established a *T. ludeni* colony from 1000 individuals collected on common bean plants *Phaseolus vulgaris* L. (Fabales: Fabaceae) in a home garden in Palmerston North, New Zealand, and reared them on bean plants grown in pots. *Phytoseiulus persimilis* was obtained from BioForce Ltd., New Zealand, and reared on *T. ludeni* before experiments. The colonies of *T. ludeni* and *P. persimilis* were separately maintained in two bioassay rooms, and the experiments were conducted under the environmental conditions of 25°C ± 1°C, 60% ± 10% RH, and 14:10 h (light:dark) photoperiod. The first expanded leaves of 1‐ to 2‐week‐old plants were used for the experiments.

### 2.2 Mite Preparation for Experiments

To obtain spider mites of specific life stages for the experiment, 10 *T. ludeni* female adults were randomly collected from the colony and transferred onto a bean leaf square (5 cm × 5 cm) placed upside down on a water‐saturated cotton pad in a Petri dish (9 cm diameter × 1 cm height). Five such Petri dishes were set up. After 24 h, the females were then moved to a new leaf square of the same size in other Petri dishes, and this process was repeated for 10 times. Newly hatched larvae were allowed to feed in situ on leaf squares and develop into the desired life stages for the experiment.

To obtain *P. persimilis* adults for the experiment, female adults collected from the breeding colony were individually transferred onto a bean leaf square (5 cm × 5 cm) placed upside down on a water‐saturated cotton pad in a Petri dish; the leaf square was previously infested with five *T. ludeni* female adults. Female adults of *P. persimilis* were allowed to lay eggs on the leaf square for 24 h and then transferred to new leaf squares infected with spider mites. The *P. persimilis* eggs were allowed to hatch and develop into adults under controlled environmental conditions. Newly emerged *P. persimilis* female adults were individually paired and mated with a male and starved for 24 h before being used for the experiment.

### 2.3 Vulnerability of *Tetranychus ludeni* to *Phytoseiulus persimilis*

In this experiment, three different life stages of *T. ludeni*, including egg, deutonymph, and newly emerged female adult, were provided to *P. persimilis* adults for choice. Fifteen *P. persimilis* adults (replicates) were individually introduced onto a leaf square (2 cm × 2 cm) placed upside down on a water‐saturated cotton pad in a Petri dish. The leaf square was infested with 15 individuals of *T. ludeni* of different stages, that is, five eggs, five deutonymphs, and five newly emerged female adults. After 24 h, the prey numbers of each stage consumed by *P. persimilis* were observed and recorded under a stereo microscope.

To assess the prey preference of *P. persimilis* , we calculated the preference index according to Manly (1974):

$$\beta_{1}=\frac{\log\frac{e_{1}}{A_{1}}}{\log\frac{e_{1}}{A_{1}}+\log\frac{e_{2}}{A_{2}}+\log\frac{e_{3}}{A_{3}}}$$

where *β*_1_ is the preference to prey type 1, *e*_1_, *e*_2_, and *e*_3_ are the remaining number of prey type 1, type 2, and type 3 after the experiment, *A*_1_, *A*_2_, and *A*_3_ are the initial number of each prey type (*n* = 5 for each prey type) presented to the predator. If the preference index is close to 1, the predator prefers prey type 1, and if it is close to 0, then other prey types are preferred.

### 2.4 Vigilance of *Tetranychus ludeni* to Olfactory Cues From Injured Conspecifics

To assess the impact of olfactory cues from injured conspecifics on the reproductive performance and survival of *T. ludeni* females, four treatments were established: (1) injured egg—a leaf arena with 10 injured conspecific eggs only; (2) injured nymph—a leaf arena with 10 injured conspecific deutonymphs; (3) injured adult—a leaf arena with 10 injured conspecific female adults; and (4) control—a leaf arena without any injured conspecifics. The leaf arena (1 cm × 1 cm) was placed upside down in the middle of a water‐saturated cotton pad in a Petri dish. The test conspecifics of the desired number and life stages were randomly collected from the colony, introduced onto the leaf arena, and killed with a dissecting needle (Gyuris et al. 2017; Ristyadi et al. 2022) to ensure that the olfactory cues were relevant to conspecifics but not to the predators.

An experimental device consisting of an olfactory cue chamber and 15 identical experimental chambers was used to conduct this experiment. The olfactory cue chamber included a Petri dish maintaining a leaf arena with one of four test olfactory cues and a transparent plastic container (8 cm diameter × 10 cm length). The structure of the experimental chambers was similar to that of the olfactory cue chamber, except for the size of the Petri dish (4.5 cm diameter × 1 cm height) and container (5 cm diameter × 8 cm length) and only one test mated female being maintained on the leaf arena (2 cm × 2 cm). For each treatment, the air from a compressed air tap was filtered through activated charcoal, measured via an airflow meter, humidified by passing through distilled water, and then blown into the olfactory cue chamber through a pipe (0.5 cm diameter). The air was then equally divided into 15 silicone pipes, each of which was connected to an experimental chamber. The air was blown out of the experimental chambers through a hole (0.5 cm diameter) at the opposite wall of the experimental chambers. The air speed was set to replace the air in the olfactory cue chamber once per minute.

The olfactory cue‐exposed females were allowed to lay eggs on the leaf arenas for 24 h, after which time they were individually transferred onto a clean leaf square of the same size in a Petri dish and exposed to the previous olfactory cue environment. Meanwhile, the olfactory cue was replaced with a new set. These procedures were repeated daily until all females died, and the longevity of those females was recorded. The number of eggs laid by the olfactory cue‐exposed females was counted daily. The size (diameter) of eggs was measured under a stereomicroscope (Leica MZ12, Germany) connected to a digital camera (Olympus SC30, Japan) and imaging software (CellSens GS‐ST‐V1.7, Olympus, Japan) installed in a computer. The egg volume was calculated as 4/3*πr* ^3^, where *r* is the radius (= diameter/2). The larvae hatching from the eggs were allowed to feed in situ and develop to protonymphs, after which they were transferred onto a clean leaf square (2 cm × 2 cm), where they developed to adults. Egg hatching rate (= number of larvae/number of eggs), and immature survival rate (= number of adults/number of larvae) were calculated, and immature developmental time was recorded. Newly emerged adults were sexed and removed from the leaf square daily.

### 2.5 Statistical Analysis

All data were analyzed using SAS 9.13 (SAS Institute Inc., USA) with a rejection level set at *α* = 0.05. Data on the number of prey eaten by predators, prey preference index, total number of eggs laid, egg size, egg hatching rate, immature survival rate, and proportion of daughters among offspring were not normally distributed (Shapiro–Wilk test), and thus analyzed using a generalized linear model (GLIMMIX Procedure): with a Poisson distribution and a log‐link function for the number of prey eaten by predators, total number of eggs laid, and egg size; with a Gamma distribution and a log‐link function for the prey preference index, and with a Binomial distribution and a logit‐link function for the egg hatching rate, immature survival rate, and proportion of daughters. An adjusted Tukey–Kramer test was applied for multiple comparisons between treatments. The egg hatching rate, immature survival rate, and proportion of daughters in relation to egg size were determined by a generalized linear model (GLIMMIX Procedure) with a Gamma distribution and a log‐link function. Data on adult survival were compared using a Wilcoxon test (LIFETEST procedure).

## 3 Results

### 3.1 Vulnerability of *Tetranychus ludeni* to *Phytoseiulus persimilis*

**FIGURE 1:**
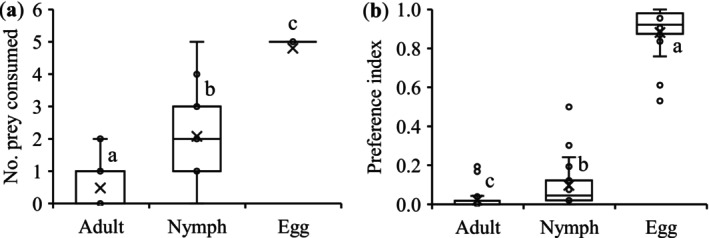
Mean (± SE) number of prey of different life stages consumed by *Phytoseiulus persimilis* (a) and preference index of *P. persimilis* on different life stages of *T. ludeni* (b). In each box plot, the box represents the interquartile range [25th (Q1) to 75th (Q3) percentiles], with the mean shown as “×,” the median as a line, and the data points as cycles. Whiskers extend from the minimum value to Q1 and from Q3 to the maximum value. Boxes with the same letters are not significantly different (*p* > 0.05).

*Phytoseiulus persimilis* significantly preferred *T. ludeni* eggs over the nymphs and adults for feeding, with an increased consumption rate of 56.7% and 90.0%, respectively (*F*_2,72_ = 36.25, *p* < 0.0001) (Figure 1a). Thus, the prey preference index of *P. persimilis* was significantly higher on *T. ludeni* eggs than on the nymphs and adults, with an increased preference index of 89.0% and 97.6%, respectively (*F*_2,72_ = 32.63, *p* < 0.0001) (Figure 1b).

### 3.2 Vigilance of *Tetranychus ludeni* to Olfactory Cues From Injured Conspecifics

**FIGURE 2:**
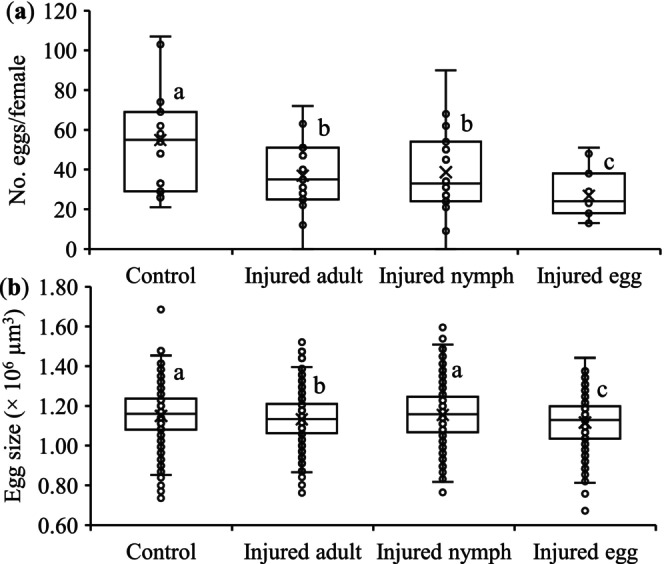
Mean (± SE) total eggs (a) and egg size (b) produced by *Tetranychus ludeni* females in response to cues from injured conspecifics of different life stages. In each box plot, the box represents the interquartile range [25th (Q1) to 75th (Q3) percentiles], with the mean shown as “×,” the median as a line, and the data points as cycles. Whiskers extend from the minimum value to Q1 and from Q3 to the maximum value. Boxes with the same letters are not significantly different (*p* > 0.05).

As shown in Figure 2a, olfactory cues from the injured conspecifics significantly suppressed oviposition, with significantly fewer eggs laid by *T. ludeni* females when they detected conspecific cues from the injured eggs (i.e., 51.2%, 32.3%, and 35.3% reduction compared to the control, injured adults, and injured nymphs, respectively) (*F*_3,54_ = 47.33, *p* < 0.0001). Egg size varied significantly between treatments, that is, females produced significantly larger eggs in control and when exposed to cues from the injured nymphs (3.2% and 3.5% larger in size compared to injured eggs, respectively), intermediate‐size eggs in response to injured adult cues (1.1% larger in size compared to injured eggs), and significantly smaller eggs when exposed to cues from injured eggs (*F*_3,2354_ = 9.83, *p* < 0.0001) (Figure 2b).

**FIGURE 3:**
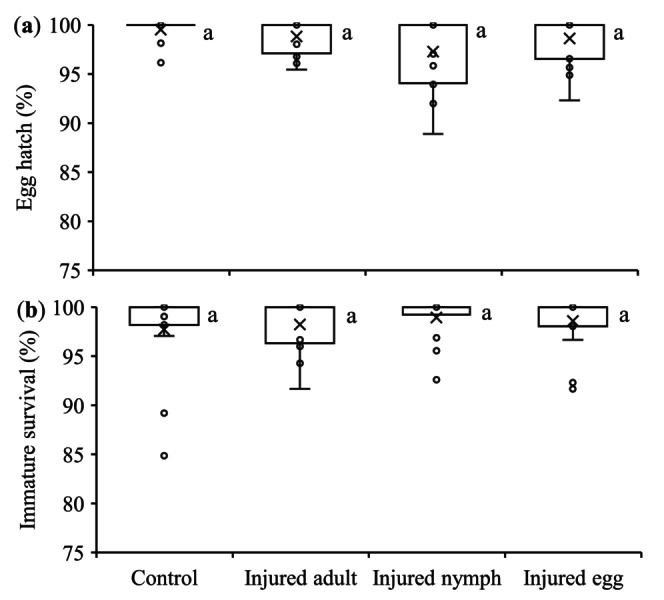
Mean (± SE) proportion of survival of eggs (a) and immature nymphs (b) produced by *Tetranychus ludeni* females in response to cues from injured conspecifics of different life stages. In each box plot, the box represents the interquartile range [25th (Q1) to 75th (Q3) percentiles], with the mean shown as “×,” the median as a line, and the data points as cycles. Whiskers extend from the minimum value to Q1 and from Q3 to the maximum value. Boxes with the same letters are not significantly different (*p* > 0.05).

The egg hatching rate varied between 97.3% and 98.8% and immature survival rate between 97.7% and 98.9%, which were not significantly affected by injured conspecific cues (*F*_3,54_ = 2.49 and 1.39 for egg hatch and immature survival, respectively, *p* > 0.05) (Figure 3).

**FIGURE 4:**
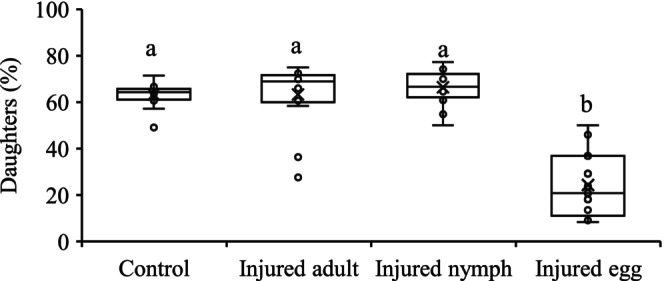
Mean (± SE) proportion of daughters produced by *Tetranychus ludeni* females in response to cues of injured conspecifics of different life stages. In each box plot, the box represents the interquartile range [25th (Q1) to 75th (Q3) percentiles], with the mean shown as “×,” the median as a line, and the data points as cycles. Whiskers extend from the minimum value to Q1 and from Q3 to the maximum value. Boxes with the same letters are not significantly different (*p* > 0.05).

The proportion of daughters among offspring was significantly lower when females detected cues from injured eggs with a reduction of 61.5%, 61.5%, and 63.3% for control, injured adults, and injured nymphs, respectively (*F*_3,54_ = 61.54, *p* < 0.0001) (Figure 4).

**FIGURE 5:**
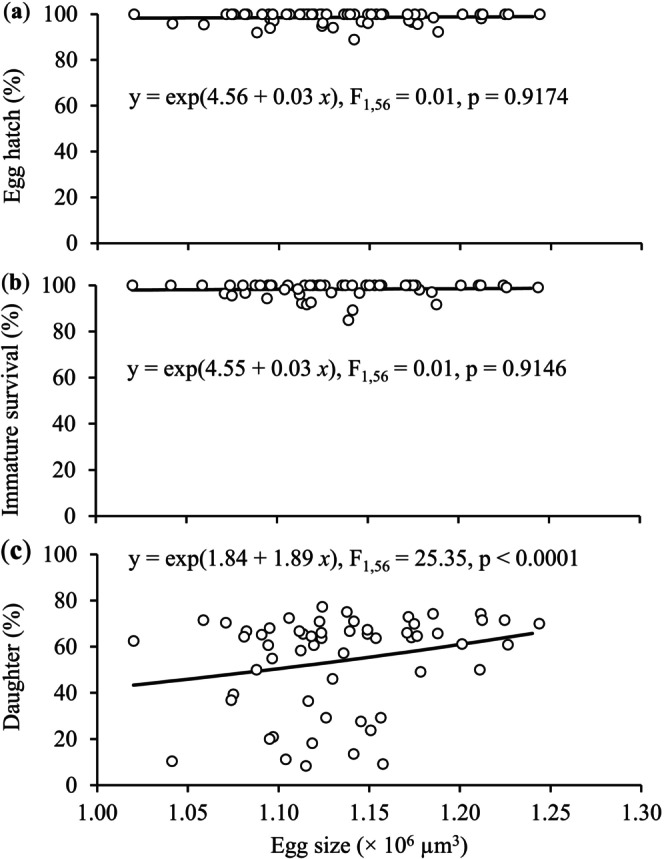
Egg hatch (a), immature survival (b), and daughters produced (c) in relation to egg size in *Tetranychus ludeni*.

The egg size had no significant effect on egg hatching and immature survival rates (*F*_1,56_ = 0.01 and 0.01 for egg hatch and immature survival, respectively, *p* > 0.05) (Figure 5a,b). However, the proportion of daughters among offspring significantly increased with increasing egg size (*F*_1,56_ = 25.35, *p* < 0.0001) (Figure 5c).

**FIGURE 6:**
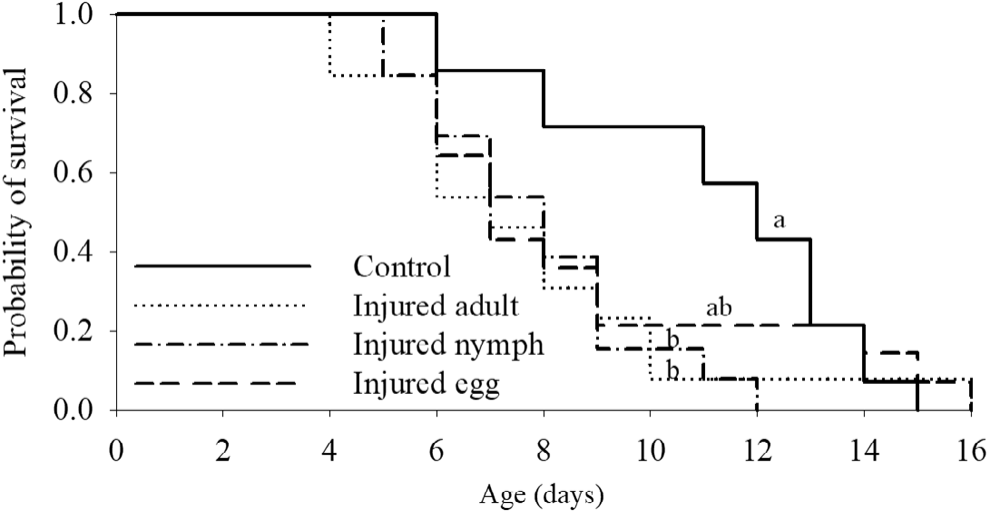
Survival of *Tetranychus ludeni* females in response to cues of injured conspecifics of different life stages. Lines with the same letters are not significantly different (*p* > 0.05).

Females had a significantly shorter longevity when they detected cues from injured nymphs and adults (7.8 and 7.7 days, respectively) compared to the control females (11.1 days), with no significant difference detected between females exposed to injured eggs (8.7 days) and control females ($x_{3}^{2}$ = 8.23, *p* = 0.0415) (Figure 6).

## 4 Discussion

Predation risk imposes profound selective pressures on prey behaviors and life‐history strategies, yet the mechanisms by which females adjust reproduction in response to stage‐specific risk signals, particularly through indirect cues from injured conspecifics, remain poorly understood. We bridge this gap in this study by demonstrating that *T. ludeni* females not only discriminate olfactory cues from vulnerable conspecific stages but also adjust reproductive investment accordingly, revealing the sophisticated interactions between maternal effects and predation risk perception.

### 4.1 Vulnerability of *Tetranychus ludeni* to *Phytoseiulus persimilis*

The preferential predation on *T. ludeni* eggs over late developmental stages (i.e., larvae, nymphs, and adults) by *P. persimilis* (Figure 1) aligns with well‐documented life stage preference patterns in phytoseiid‐tetranychid systems (Grafton‐Cardwell et al. 1997; Moghadasi et al. 2013; Blackwood et al. 2001; Badii et al. 2004; Furuichi et al. 2005; Ganjisaffar and Perring 2015; Jyothis and Ramani 2024). This life stage‐specific vulnerability of spider mites may likely arise from the interactions of ecological and physiological factors. First, as a blind predator (van Wijk et al. 2008), phytoseiids rely on tactile and chemical cues during foraging, making immobile eggs easier to locate and handle than other life stages (Grafton‐Cardwell et al. 1997; Messelink et al. 2006; Kasap and Atlihan 2011; Moghadasi et al. 2013; Soleymani et al. 2016). Second, prey eggs represent a nutrient‐rich resource due to yolk provisioning for embryonic development (McMurtry 1987; Xiao et al. 2013). Gagné et al. (2002) revealed that in a ladybird beetle *Coleomegilla maculata lengi* Thimberlake, egg cannibalism benefits predators with higher energetic returns per unit handling time. Third, eggs lack the behavioral defenses (e.g., escape responses) and physical protections (e.g., hardened cuticles) of nymphs and adults, reducing risk to predators (Lingle et al. 2008; Kasap and Atlihan 2011; Choh et al. 2012; Giachetti et al. 2022). The strong preference for eggs (Figure 1b) suggests that *P. persimilis* employs an optimal foraging strategy, maximizing energy and nutrient intake while minimizing predation costs, a phenomenon observed across predatory arthropods (Grafton‐Cardwell et al. 1997; Gagné et al. 2002; Messelink et al. 2006). The life stage‐specific vulnerability in spider mites emerges from an evolutionary arms race, where predator foraging efficiency interacts with prey life‐history traits.

### 4.2 Vigilance of *Tetranychus ludeni* to Olfactory Cues From Injured Conspecifics

Chemical communication serves as a fundamental mechanism for risk assessment in predator–prey interactions (Tollrian and Harvell 1999; Dicke and Grostal 2001), while the ecological and evolutionary consequences of such chemical detection systems remain poorly understood (Grostal and Dicke 1999; Ristyadi et al. 2022). Our study reveals two key findings that advance this understanding: (1) *T. ludeni* females significantly reduced lifetime fecundity when exposed to olfactory cues from injured conspecifics, and (2) this response was significantly stronger when cues originated from injured eggs compared to deutonymphs or adults (Figures 2a and 6). These results demonstrate that, in addition to the direct predator cues (Ristyadi et al. 2022), *T. ludeni* could also respond to the indirect infochemicals from injured conspecifics, confirming similar findings in congeneric species (Grostal and Dicke 1999; Oku, Yano, Osakabe, and Takafuji 2003; Oku et al. 2004; Azandémè‐Hounmalon et al. 2016; Gyuris et al. 2017; Tscholl et al. 2023). Importantly, females showed life stage‐specific sensitivity, with heightened vigilance to cues from the most vulnerable life stage (eggs), suggesting they can assess differential predation risks based on cue source. This aligns with *P. persimilis* 's strong egg preference (Figure 1) and supports the theory of risk‐sensitive reproductive strategies, where prey adjust investment based on perceived threats to offspring survival (Grostal and Dicke 1999; Fievet et al. 2008; Hua et al. 2014; Britton and Ballentine 2020; Corbel and Carazo 2022).

A classical life‐history theory predicts trade‐offs between reproduction and survival under resource limitation (Roff 1992; Stearns 1998; Zera and Harshman 2001; De Loof 2011; Martin et al. 2007; Blacher et al. 2017; Cingolani et al. 2020). However, challenging this paradigm, we observed simultaneous reductions in both fecundity and longevity (Figures 2a and 6) as reported in *T. urticae* (Li and Zhang 2019). These results suggest a stress‐mediated response where perceived predation risk triggers broad physiological changes rather than strategic resource reallocation. Interestingly, egg‐derived cues elicited particularly strong effects, reducing fecundity by 47.6%–54.6% without corresponding longevity trade‐offs (Figures 2a and 6). Such non‐consumptive effects may significantly impact population dynamics, potentially rivaling the effects of direct predation (Preisser et al. 2005; Zanette et al. 2011; MacLeod et al. 2014; Buchanan et al. 2017; Pessarrodona et al. 2019). In biological control contexts, such stress responses to conspecific injury cues could substantially enhance pest management efficacy beyond direct prey consumption.

Egg size, determining the initial resource for embryonic development (Fox et al. 1994), typically correlates with offspring fitness. Larger eggs generally have a higher hatching rate (Goulden et al. 1987; Saino et al. 2004; Amiri et al. 2020) and produce larger and more viable offspring (Fox et al. 1994; Arnold et al. 2006; Macke, Magalhaes, et al. 2011; Song et al. 2020). In this study, egg size had no significant effect on egg hatch and immature survival (Figure 3), suggesting that females partitioned sufficient nutrients across all eggs regardless of size. Alternatively, when future environmental conditions are uncertain or unpredictable, egg size may not be a reliable indicator of offspring fitness (Karlsson and Wiklund 1985; Lalonde 2005; Morrongiello et al. 2012; Weerawansha et al. 2022b, 2023). However, it is noted that our experimental design did not allow offspring to be exposed to the conspecific injury cues during development; future studies should examine how such cues interact with egg size effects across life stages.

In haplodiploid arthropods, such as spider mites (Macke, Magalhaes, et al. 2011; Macke, Magalhães, et al. 2011; Macke et al. 2012, 2014; Weerawansha et al. 2022a, 2022b) and thrips (Katlav et al. 2021), females tend to fertilize larger eggs that develop to diploid daughters. We show evidence of females laying larger eggs and producing more daughters in treatment of injured deutonymphs and control (Figures 2b and 4), and a positive relationship between the overall proportion of daughters and egg size (Figure 5). The latter agrees with the results of previous studies testing the sex allocation of *T. ludeni* under varying social environments (Weerawansha et al. 2022a, 2022b, 2023). We further reveal that when perceived cues from injured female adults, females laid smaller eggs but produced a similar high proportion of daughters as the control (Figures 2b and 4). In spider mites, female is the sex that tends to disperse to search for new habitats due to food deficiency or habitat deterioration (Azandémè‐Hounmalon et al. 2016; Schausberger et al. 2021; Zhou et al. 2021b). Therefore, our results suggest that *T. ludeni* females could manipulate offspring sex ratio by fertilizing more smaller eggs with more daughters produced, because cues from the injured conspecific females implied a high potential for immediate mortality of ovipositing females; producing more dispersing daughters could be a bet‐hedging strategy to enhance colony persistence under the high predatory stress (Schausberger et al. 2021). Such plasticity in sex allocation demonstrates the sophisticated risk assessment capabilities of *T. ludeni* and highlights how indirect predation cues can shape reproductive strategies in unexpected ways.

## 5 Conclusion

Our study demonstrates that *P. persimilis* preferentially preyed on *T. ludeni* eggs, indicating their high vulnerability compared to deutonymphs and adults. Ovipositing *T. ludeni* females exhibited stage‐specific antipredator strategies in response to olfactory cues from injured conspecifics, significantly reducing reproductive output when perceiving olfactory cues from injured eggs, the life stage most targeted by predators. Thus, females might assess predation risk not only based on conspecific cues but also their relevance to offspring vulnerability. While egg size did not affect hatching or immature survival in the absence of predators, future studies should test whether these traits are compromised under direct predatory stress. Notably, females adjusted offspring sex ratios under perceived risk, producing more dispersive daughters when exposed to cues from injured adults, which could be a potential bet‐hedging strategy to enhance colony persistence. Our study bridges the vulnerability of prey conspecific stages to predators with prey vigilance in response to indirect predatory signals from those stages. It also highlights the mechanisms of females adjusting their reproductive investment under varying predation risks. However, under natural conditions, prey may perceive both direct and indirect predator cues simultaneously (Walzer and Schausberger 2013). Future research should integrate both cue types to evaluate whether one amplifies the impact of the other on prey survival and reproduction, which is essential for a comprehensive understanding of antipredator strategies of prey in nature.

## Author Contributions

**Resona Simkhada:** conceptualization (lead), data curation (lead), formal analysis (equal), investigation (lead), methodology (lead), validation (equal), writing – original draft (lead), writing – review and editing (lead). **Jhaman Kundun:** conceptualization (equal), investigation (supporting), methodology (supporting), writing – review and editing (equal). **Svetla Sofkova‐Bobcheva:** conceptualization (equal), supervision (supporting), writing – review and editing (equal). **Xiong Zhao He:** conceptualization (lead), data curation (equal), formal analysis (equal), methodology (lead), supervision (lead), validation (equal), writing – review and editing (lead).

## Conflicts of Interest

The authors declare no conflicts of interest.

## Supporting information


**Data S1:** ece372257‐sup‐0001‐DataS1.xlsx.

## Data Availability

Data were uploaded as Supporting Information.
